# Extranodal Rosai–Dorfman disease: a rare soft tissue neoplasm masquerading as a sarcoma

**DOI:** 10.1186/1477-7819-11-63

**Published:** 2013-03-09

**Authors:** Mahathi Komaragiri, Lauren S Sparber, Maria Laureana Santos-Zabala, Michael Dardik, Ronald S Chamberlain

**Affiliations:** 1 Saint George’s University School of Medicine, University Centre, Grenada, West Indies; 2 Department of Surgery, Saint Barnabas Medical Center, 94 Old Short Hills Road, Livingston, NJ, 07039, USA; 3 Department of Pathology, Saint Barnabas Medical Center, 94 Old Short Hills Road, Livingston, NJ, 07039, USA; 4 Department of Surgery, University of Medicine and Dentistry of New Jersey, 185 South Orange Avenue, Newark, NJ, 07013, USA

**Keywords:** Rosai–Dorfman disease soft tissue, Rosai–Dorfman disease cutaneous, Sinus histiocytosis with massive lymphadenopathy

## Abstract

**Introduction:**

Rosai–Dorfman disease (RDD) is a rare proliferative histiocytic disorder of unknown etiology. RDD typically presents with generalized lymphadenopathy and polymorphic histiocytic infiltration of the lymph node sinuses; however, occurrences of extranodal soft tissue RDD may rarely occur when masquerading as a soft tissue sarcoma.

**Materials and methods:**

A comprehensive search of all published cases of soft tissue RDD without associated lymphadenopathy was conducted using PubMed and Google Scholar for the years 1988 to 2011. Ophthalmic RDD was excluded.

**Results:**

Thirty-six cases of extranodal soft tissue RDD, including the current one, have been reported since 1988. Anatomical distribution varied among patients. Four (11.1%) patients presented with bilateral lesions in the same anatomic region. Pain was the most common symptom in six (16.8%) patients. Sixteen (41.6%) patients were managed surgically, of which one (2.8%) case experienced recurrence of disease.

**Conclusion:**

RDD is a rare inflammatory non-neoplastic process that should be considered in the differential diagnosis of a soft tissue tumor. Thus, differentiation of extranodal RDD from more common soft tissue tumors such as soft tissue sarcoma or inflammatory myofibroblastic tumor is often difficult and typically requires definitive surgical excision with histopathological examination. While the optimal treatment for extranodal RDD remains ill-defined and controversial, surgical excision is typically curative.

## Background

Sinus histiocytosis with massive lymphadenopathy (SHML) is a class II histiocytosis first described as a unique clinicopathologic entity by Rosai and Dorfman in 1969 [1]. Although lymph nodes are more commonly involved, any organ may be affected – thus the term RDD has been adopted in place of SHML [1]. A rare disease, RDD is distributed worldwide, predominantly affecting young people and with a slight male predominance [2]. The disease typically presents with bilateral painless lymphadenopathy of the head and neck as well as fever, leukocytosis, elevated erythrocyte sedimentation rate and polyclonal hypergammaglobulinemia [3,4]. Extranodal presentations have also been described, with the most common sites including skin and nasal sinuses [4]. RDD is considered a non-neoplastic manifestation with a self-limited course; however, it may also undergo exacerbations and remissions rendering treatment to be necessary [2]. Histologically, RDD classically shows an inflammatory infiltrate rich in lymphocytes, plasma cells and large histiocytes [4]. RDD histiocytes are unique because they phagocytose intact lymphocytes and other immune cells, a histological hallmark of the disease termed emperipolesis [5]. The largest report of RDD (1969) involved 423 cases, with 182 patients having extranodal disease [3]. Only 13 patients in this series presented with soft-tissue RDD without detectable lymphadenopathy [3]. Here we describe an unusual case of RDD in a middle-aged African American female presenting as a painful right medial thigh mass.

## Case presentation

**Figure 1:**
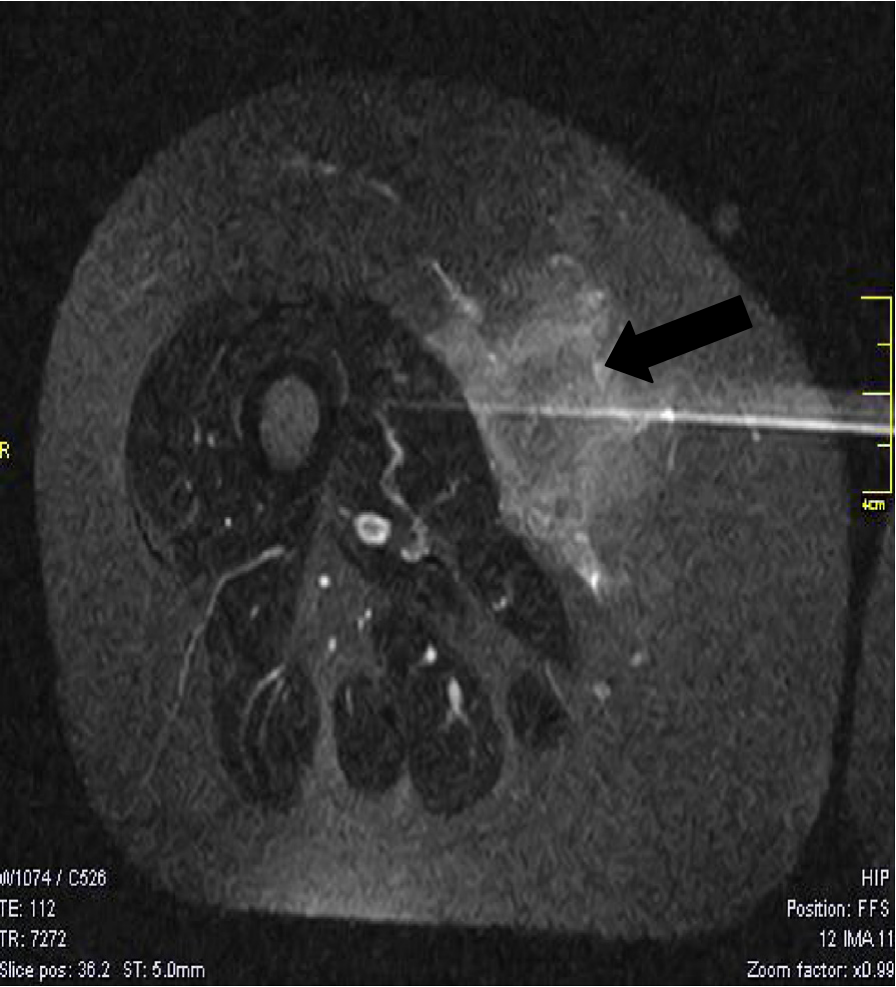
**Large radiolucent mass in the right medial thigh.** T1-weighted transverse magnetic resonance image of the right lower extremity demonstrating a large radiolucent mass in the right medial thigh (black arrow).

A 56-year-old African American female presented to the Saint Barnabas Medical Center (Livingston, NJ, USA) with a 1-year history of an enlarging painful right medial thigh mass. Her medical history was significant for hypertension, diabetes, hypercholesterolemia, degenerative disk disease and asthma. The mass was noted to be extremely painful and was located anterior and superficial to the adductor muscle group in the right medial thigh. The patient reported a pulling, painful sensation in the knee joint as well as discomfort upon standing for long periods of time. She denied any constitutional symptoms and had no reported neurological deficits. No weight loss was reported. A chest, abdomen, and pelvis computed tomography scan was performed, demonstrating no systemic adenopathy. A magnetic resonance imaging study of the right lower extremity was obtained and revealed a 7 cm × 5 cm × 3.2 cm ill-defined, enhancing soft tissue mass, suspicious for a soft tissue sarcoma located in the deep subcutaneous tissues of the right thigh, adjacent to the vastus medialis muscle (Figure 1). The lesion was suspicious for a primary malignancy/sarcoma and the patient underwent a core biopsy of the lesion. Biopsy results showed an inflammatory mass most consistent with an inflammatory myofibroblastic tumor. However, the tissue sample was limited and a diagnosis of sarcoma could not be excluded. An incisional biopsy was then completed that demonstrated an inflammatory pseudotumor with mesenchymal fatty and fibroblastic proliferation and prominent inflammatory cell infiltration. There was no evidence of any atypia or identifiable lymphoma. The mass was positive for Vimentin and BCL-2, and was negative for all other markers including desmin, smooth muscle actin, CD34, AE1, S-100, p53 ALK-1 and EBER-RNA. The patient’s symptoms progressed and a wide local excision of the mass was completed.

**Figure 2:**
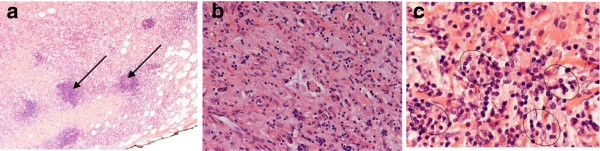
**Microscopic examination of right medial thigh mass demonstrating characteristic emperipolesis. (a)** Groups of lymphoid aggregates (black arrow) and scattered pale areas of fibroadipose tissue (H & E stain, original magnification ×10). **(b)** Histiocytes and multinucleated cells among mixed inflammatory cells that include plasma cells and lymphocytes (H & E stain, original magnification ×20). **(c)** Histiocytes engulfing lymphocytes and plasma cells (emperipolesis) (H & E stain, original magnification ×40).

**Figure 3:**
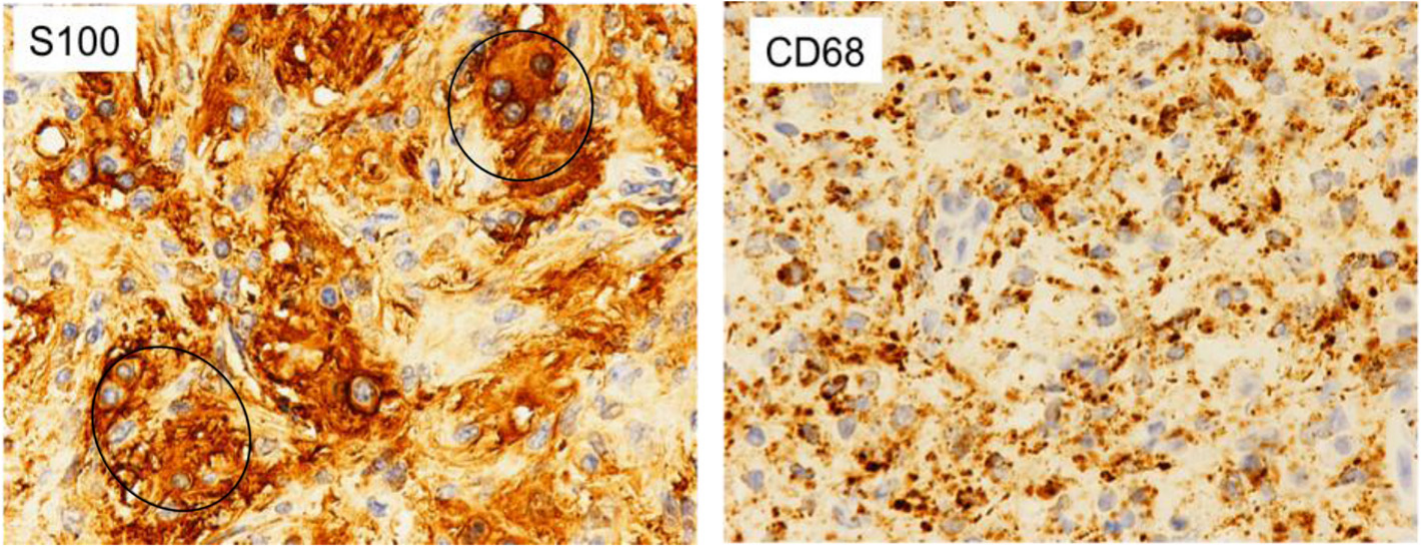
**Immunohistochemical staining of the right medial thigh mass.** Immunohistochemical stains are positive for **(a)** S-100 and **(b)** CD68 (original magnification ×20).

On gross examination, the specimen weighed 357 g. There was an ill-defined, firm, yellow to tan mass deep within the subcutaneous tissue that measured 8 cm × 7.5 cm × 6 cm. The mass had a fleshy cut surface. There were no overlying skin changes. Microscopic examination demonstrated a mixed inflammatory background including lymphocytes, plasma cells, polymorphonuclear leukocytes and small areas of bland fibroblasts (Figure 2a,b). There were large aggregates of pale-staining histiocytes demonstrating emperipolesis (Figure 2c). Immunohistochemical stains for S-100 and CD68 were strongly positive (Figure 3a,b) in the histiocytes. Fluorescence *in situ* hybridization was negative for MDM2 gene amplification, excluding a well-differentiated liposarcoma. The above immunophenotype and characteristic histological findings of emperipolesis were consistent with a final pathologic diagnosis of extranodal RDD.

Postoperatively the patient had experienced persistent but resolving right medial thigh pain and was referred for physiotherapy. At the patient’s 9-month follow-up, there was no recurrence of the tumor on the right side and the painful symptoms were resolving. However, she then presented with a relatively nontender 2 cm × 4 cm cyst-like mass on the left medial thigh in the popliteal region. Magnetic resonance imaging demonstrated a 5.3 cm × 4 cm × 1.6 cm area consistent with either a lymphangioma or resolving necrosis from a residual mass. Core biopsy results yielded extranodal RDD with cells positive for S-100, CD68 and CD117. The sample was negative for AE1/3 and CD34. CD1a testing was not performed on the core biopsy. An excisional biopsy was performed and on gross examination the specimen was a fragment of tan–yellow adipose tissue measuring 7 cm × 6 cm × 1.5 cm, with a poorly defined tan-colored region of induration measuring 2 cm × 1.2 cm × 1 cm. Microscopic examination was once again consistent with extranodal RDD confirmed with immunohistochemical stains (positive for S-100 and CD68). CD1a immunohistochemical staining was not performed on the excisional biopsy tissue.

## Results

A comprehensive search of all published cases of soft tissue RDD without associated lymphadenopathy was conducted using PubMed and Google Scholar for the years 1988 to 2011. Ophthalmic RDD was excluded. Among reported cases of Rosai–Dorfman tumors, 43% of patients had extranodal disease with associated lymphadenopathy; however, only 3% of the patients had soft tissue RDD without detectable lymph node involvement [3,5,6]. Overall, 36 cases of extranodal soft tissue RDD had been documented since 1969, including the current case. Clinical and treatment data for all reported soft tissue RDD are detailed in Table 1. Among these 36 patients, 17 (47.2%) patients were male and 19 (52.7%) patients were female (male:female ratio, 0.89:1). The overall mean age was 45.3 years (range 10 months to 72 years), with a mean age for males and females of 45.8 and 47.0 years, respectively. The most common anatomic location for extranodal soft tissue RDD was the lower extremity (38.9%), followed by the upper extremity (36.1%), the torso (36.1%) and the head and neck (19.4%). Many patients (41.6%) presented with multiple lesion sites, with much fewer (11.1%) presenting with bilateral lesions in the same anatomic region. Pain was the most commonly reported symptom (16.8%). Surgical resection was described in 16 cases (41.6%), of which only one case (2.8%) experienced recurrence of disease. The remaining 19 cases (52.8%) were managed medically with dapsone, steroids or observation. Of these patients, some patients (11.1%) experienced spontaneous resolution, other cases (13.9%) had partial regression, while no changes were observed in seven (19.4%) cases and one patient (2.8%) experienced regrowth of the tumor. Of the reported cases, three patients (8.3%) had no reported follow-up while one case (2.8%) did not report treatment. Among cases in which immunohistochemistry was performed, 34 cases (94.4%) were reactive to S-100 stain and 30 cases (83.3%) were reactive to CD68 stain.

**Table 1:** Results of all published reports of extranodal soft tissue Rosai Dorfman disease (1988 to 2012)

**Case**	**Study**	**Location**	**Age (years)**	**Sex**	**Presenting symptoms**	**IHC**	**Surgical excision**	**Status after diagnosis and treatment**
1	Suster et al, 1988 [7]	Lateral upper right arm and middle third of left thigh	72	F	Multiple firm flesh-colored subcutaneous nodules	S-100+	No	7 years; spontaneous resolution
2	Rasool et al, 1996 [8]	Right index finger	10 mo	F	Right index finger swelling, pain, axilla, axillary lymph node	NR	Yes	14 months, no recurrence
3	Govender et al, 1997 [9]	Chest wall	34	F	Superficial firm mass	S-100+	Yes	6 months, no recurrence
						CD68+		
4	Child et al, 1998 [10]	Posterior left thigh	36	F	Hyperpigmented indurated plaque with multiple nodules, occasional pain	S-100+	No	12 months; no new lesions, new nodules within plaque
						CD68+		
5	Quaglino et al, 1998 [11]	Skin of arms and buttocks	70	F	Nodules on arms, face, buttock	S-100+	NR	NR
						CD1a–		
6	Huang et al, 2001 [12]	Medial right upper arm	43	F	Numbness and paresthesias of forearm and wrist	S-100+	Yes	12 months; no recurrence, mild hypoesthesia over lesion site
						CD68+		
7	Stefanato et al, 2002 [13]	Upper back, thighs, feet	55	F	Firm reddish brown dome-shaped papules with a single episode of belpharoconjunctivitis	S-100+	No	24 months; spontaneous resolution
						CD1a–		
8	Yoon et al, 2005 [5]	Left ankle, right forearm and arm	36	M	Nontender mobile nodules, left ankle pain, epistaxis	S-100+	Yes	4 months; recurrence in right maxillary sinus
9	Kong et al, 2007 [1]	Upper right arm	55	M	Flat-topped papules and nodules	S-100+	No	55 months; spontaneous resolution
						CD68+		
10	Kong et al, 2007 [1]	Left thigh and back	65	M	Brown plaque and nodules	S-100+	No	31 months; partial regression
						CD68+		
11	Kong et al, 2007 [1]	Left thigh	49	F	Red to brown nodules, tenderness	S-100+	No	31 months; partial regression
						CD68+		
12	Kong et al, 2007 [1]	Left chest	51	M	Pinkish papules	S-100+	Yes	28 months; no recurrence at original site but appearance of papules in popliteal area
						CD68+		
13	Kong et al, 2007 [1]	Left upper back	52	M	Red to dark-red papules, pruritis	S-100+	No	27 months; spontaneous resolution
						CD68+		
14	Kong et al, 2007 [1]	Lower back	45	M	Clusters of pinkish papules	S-100+	Yes	26 months; no recurrence
						CD68+		
15	Kong et al, 2007 [1]	Upper left arm	52	F	Red to brownish plaque with scattered papules and nodules	S-100+	Yes	8 months, no recurrence
						CD68+		
						CD1a+		
16	Kong et al, 2007 [1]	Right thigh and left calf	55	M	Hyperpigmented infiltrated plaque with papules surrounding	S-100+	No	24 months; persistence
						CD68+		
17	Kong et al, 2007 [1]	Left arm and right thigh	70	F	Erythematous papuloplaques with papulovesicles and pustules, pruritis	S-100+	No	24 months; partial regression
						CD68+		
18	Kong et al, 2007 [1]	Left arm and sacral back	48	M	Pinkish to red papules	S-100+	No	NR
						CD68+		
19	Kong et al, 2007 [1]	Left thigh	49	F	Brownish indurated plaque with satellite papules	S-100+	No	24 months; persistence
						CD68+		
20	Kong et al, 2007 [1]	Left thigh and back	43	M	Confluent papules and erythematous plaque	S-100+	Yes	7 months; no recurrence
						CD68+		
21	Kong et al, 2007 [1]	Right thigh	45	M	Plane hyperpigmented plaque	S-100+	Yes	15 months; no recurrence
						CD68+		
22	Kong et al, 2007 [1]	Suprasternal fossa and sacral back	56	F	Two dome-shaped, exophytic masses surrounded by few small papules, ulceration formed in one lesion	S-100+	Yes	15 months; no recurrence
						CD68+		
23	Kong et al, 2007 [1]	Right upper back	47	M	Single dark-red nodule	S-100+	Yes	14 months; no recurrence
						CD68+		
24	Kong et al, 2007 [1]	Abdomen and right buttock	21	M	Confluent papules and infiltrated plaque dotted with brownish papules	S-100+	No	12 months; slowly growing
						CD68+		
25	Kong et al, 2007 [1]	Upper right arm	42	M	Grouped pinkish papules, pain of wrist and shoulder	S-100+	No	11 months; persistence
						CD68+		
26	Kong et al, 2007 [1]	Face, buttock, abdomen and bilateral lower extremities	22	F	Multiple coalescing nodules, erythematous patches and plaques, some with tumorous appearance, pruritis	S-100+	No	NR
						CD68+		
27	Kong et al, 2007 [1]	Cheek, back and buttock	54	M	Dark-red and brownish nodules	S-100+	No	NR
						CD68+		
28	Kong et al, 2007 [1]	Upper left arm	52	F	Single subcutaneous mass, fever	S-100+	Yes	9 months, no recurrence
						CD68+		
29	Kong et al, 2007 [1]	Right cheek	40	M	Erythematous papuloplaque	S-100+	No	6 months; partial regression
						CD68+		
30	Kong et al, 2007 [1]	Right cheek	52	F	Erythematous papuloplaque	S-100+	No	5 months; persistence
						CD68+		
31	Kong et al, 2007 [1]	Left cheek and neck	38	M	Erythematous papuloplaque	S-100+	No	2 months; persistence
						CD68+		
32	Penna Costa et al, 2009 [3]	Left paravertebral mass in posterior mediastinum	49	F	Dyspnea and cough and cervical lymphadenopathy	S-100+	Yes	12 months; no recurrence
						CD68+		
						CD1a–		
33	Potts et al, 2008 [2]	Right forearm	31	F	Firm, hyperpigmented mass	NR	Yes	8 months; recurrence
								7 months after re-excision; no recurrence
34	Molina-Garrido et al, 2011 [14]	Parieto-occipital cutaneous lesion	43	M	Red–yellow nodule, pain in right inferior maxillary area	S-100+	Yes	3 months; no recurrence
						CD68+		
						CD1a–		
						CD20–		
35	Shi et al, 2011 [15]	Face, neck extremities	45	F	Nonpruiginous papulonodular plaques	S-100+	No	5 weeks; partial regression
						CD68+/–		
						CD1a–		
36	Current study	Medial right thigh; Medial left thigh	56	F	Enlarging mass, knee joint pain	S-100+	Yes	9 months; no recurrence on right but new lesion on left
						CD68+		

Totals: Mean age 46.3 years (10 months to 72 years); 17 male (M):19 female (F). Surgical excision: 16 yes, 19 not reported (NR).

IHC, immunohistochemistry; S-100, protein 100% soluble in ammonium sulfate; CD68, cluster of differentiation 68; CD1a, cluster of differentiation 1a; CD20, cluster of differentiation 20.

## Discussion

A class II histiocytosis, RDD was first described as a distinct clinicopathologic entity by Rosai and Dorfman in 1969 [1]. A rare disease, RDD is distributed worldwide with 80% of cases occurring in children and young adults [16]. RDD exhibits a slight male predominance (58%) and a general predilection for individuals of African descent [16]. The largest study of RDD was conducted by Foucar, Rosai and Dorfman in 1990 and included 423 cases with a histopathological diagnosis of RDD [3].

RDD is of unknown etiology, although viral agents such as human herpes virus-6 and Epstein–Barr virus are thought to play a role in the pathogenesis via immune system dysregulation [17,18]. Levine and colleagues detected human herpes virus-6 via *in situ* hybridization in seven of nine SHML cases, while Luppi and colleagues had demonstrated human herpes virus-6 antigen expression by abnormal histiocytes [19,20]. Both sets of data suggested a causative role for human herpes virus-6 virus. Levine and colleagues also detected Epstein–Barr virus DNA by *in situ* hybridization; however, this was detected in only one of nine SHML cases, suggesting that Epstein–Barr virus infection is probably not causative but may be a contributing factor to the development of RDD [9]. Yoon and colleagues had theorized that the initiation of monocyte colony-stimulating factor-mediated histoproliferation in RDD is an abnormal reaction of the hematolymphoid system to infection, leading to a high level of immune activation and subsequent cytosis [5]. However, the pathogenesis of RDD is still poorly understood but is likely multifactorial, with many of the documented patients having a variety of coexisting immunologically mediated disorders such as asthma, systemic lupus erythematosus, rheumatoid arthritis and hemolytic anemia [2,21].

RDD is classified as either nodal or systemic (cutaneous, respiratory and/or osseous). RDD typically presents insidiously with generalized lymphadenopathy and a polymorphic histiocytic infiltration of the lymph node sinuses. The cervical lymph nodes are most commonly affected, followed by inguinal, axillary and mediastinal lymph node basins [18]. RDD may mimic a more malignant prognosis; however its clinical course varies from spontaneous regression to progressive lymphadenopathy and prolonged phases of stable disease [18,22]. Although a rare complication, death is usually a result of nodular expansion into vital organs with interference of normal organ function. Complications leading to death are otherwise not well described among existing reports [18].

Systemic RDD is more prevalent and is characterized by tumors at other sites such as bone, upper respiratory tract, skin and retro-orbital tissue [23]. The cutaneous form involves only the skin and adjacent soft tissue without associated involvement of lymph nodes or other organs [2]. The cutaneous form occurs in one-third of cases, with skin and head and neck being the most commonly affected sites [17].

The differential diagnosis for RDD is challenging and is based on clinical features as well as immunohistological analysis and radiographic features. Lymph nodes tend to be hypermetabolic and positive on positron emission tomography. Upwards of 80% of patients demonstrate polyclonal hypergammaglobulinemia and as many as 65% have a hypochromic or normochromic normocytic anemia [16]. Histologically, RDD is characterized by an accumulation of proliferating histiocytes primarily in the sinusoids of lymph nodes [5]. RDD histiocytes phagocytose intact lymphocytes and other immune cells, leading to the disease’s histological hallmark finding of emperipolesis [5]. Immunohistochemically, RDD is typically positive for S-100 and CD68 antigens and negative for CD1a antigens [24]. However, it is important to note that CD1a reactivity is more typical of Langerhans cell histiocytosis (LCH) while S-100 reactivity is more characteristic of RDD [6]. While computed tomography and magnetic resonance imaging are not diagnostic of RDD, they can exclude other possible diagnoses as well as being useful to assess local disease extension [18].

The differential diagnoses of RDD-type lesions include lymphoreticular malignancies when cervical lymphadenopathy is present or soft tissue sarcomas when patients present with extranodal disease. Table 2 details a comparison of the demographics, presenting symptoms, and histological appearance of RDD, LCH, inflammatory myofibroblastic tumor and soft tissue sarcoma. A lack of cytologic atypia typically dispels more malignant diagnoses, and immunohistochemistry profiles will demonstrate a macrophage-induced histiocytosis with emperipolesis [28]. It is also important to clearly distinguish RDD-similar S-100-positive histiocytoses such as malignant histiocytosis and LCH [1]. Malignant histiocytosis demonstrates marked cytologic atypia as well as high mitotic activity, while LCH tends to be CD1a positive with microscopic evidence of Birbeck granules [1]. In addition, neither malignant histiocytosis nor LCH demonstrate the hallmark finding of emperipolesis [1]. The more rare reticulohistiocytoma may be S-100-positive; however, this disease shows prominent ground glass appearance, abundant periodic acid Schiff-positive stain and fewer inflammatory cells in the background. These marker-specific differences are useful in providing a definitive diagnosis.

**Table 2:** Clinical features of Rosai-Dorfman disease and other common and uncommon soft tissue tumors

	**Rosai-Dorfman disease [**[6]**]**	**Langerhans cell histiocytosis [**[25]**]**	**Inflammatory myofibroblastic tumor [**[26]**]**	**Soft tissue sarcoma [**[27]**]**
Incidence (cases/million persons/year)	Rare	0.5 to 5.4	Not reported	11,280/3,000,000
Male:female ratio	1.33:1	2:1	Not reported	1:1
Racial predilection	African American	Caucasian	Not reported	None
Most common age range at diagnosis	20.6 years	0 to 15 years	6 to 10 years	<21 years
Anatomic location	Cervical lymph nodes > skin > upper respiratory tract, bone	Lymph nodes, liver spleen, skin, bone marrow, lungs > gastrointestinal tract, central nervous system	Lung > abdomen, mesentery	Lower extremity > trunk > upper extremity, retroperitoneum > head and neck > mediastinum
Lymph nodes				
Soft tissue				
Organ systems				
Symptoms at presentation	Nodal disease: massive lymphadenopathy	Fever, weight loss, lethargy, bone pain, skin and scalp erythematous rash, hepatosplenomegaly, respiratory distress	Fever, weight loss, pain, malaise, night sweats, reactive lymphadenopathy, tumor compressive symptoms	Asymptomatic mass, palpable abdominal mass with symptoms such as fullness, early satiety and vague abdominal pain
	Extranodal disease: tumors in skin, upper respiratory tract, bone with or without lymphadenopathy			
	Both: fever, weakness, weight loss, anemia, shortness of breath, headaches, nosebleeds			
Pathology	Capsular lesion with large nuclei, inflammatory infiltrate rich in lymphocytes, plasma cells and large histiocytes demonstrating emperipolesis	Noncapsulated lesion with small nuclei, eosinophils and Langerhans cells with distinct cell margins and pink granular cytoplasm with Birbeck granules	Nonencapsulated lesion containing spindle cells proliferating in a background of fibrosis, with lymphocytes, plasmacytes, histiocytes, foamy macrophages, and occasionally eosinophils and neutrophils	Highly variable location and history dependent
Immunohistochemistry	CD1a–, S-100+, CD68+, CD63+, emperipolesis	CD1a+, S-100+, CD54+, CD58+, no emperipolesis	Smooth muscle actin+, vimentin+, factor XIIIa+, S-100–	Neurofibrosarcoma: S-100 positive
				Angiosarcoma: Factor XIIIa positive
				Rhabdomyosarcoma: Myoglobin positive
Long-term prognosis	Self-limited with resection > medical therapy	Self-limited course, some variants show chronicity, patients <2 with disseminated disease are >50% likely to die	Benign, reactive, recurrent, multifocal	Malignant; course dependent on size, grade, location

S-100, protein 100% soluble in ammonium sulfate; CD68, cluster of differentiation 68; CD1a, cluster of differentiation 1a; CD63, cluster of differentiation 63; CD54, cluster of differentiation 54; CD58, cluster of differentiation 58.

Soft tissue RDD is particularly challenging to diagnose since it is often difficult to discern the exact morphology of soft tissue samples. Extranodal soft tissue RDD usually demonstrates a spindled morphology with abundant collagen deposition resulting in the hallmark emperipolesis to become more inconspicuous [6]. Furthermore, the whorled pattern that is typically seen in soft tissue RDD can also mislead clinicians to diagnose either benign or malignant fibrohistiocytosis [6]. In general, however, cells of soft tissue fibrohistiocytic lesions, such as benign fibrous histiocytoma or dermatofibrosarcoma protuberans, usually have a higher nuclear to cytoplasmic ratio, more hyperchromatic nuclei and a more distinctive whorled pattern than those of soft tissue RDD [6].

Due to the rarity of the disease and its self-limited course, no treatment protocol has been established for RDD [18]. In symptomatic cases where the disease does not resolve spontaneously, surgical excision is typically performed. Symptomatic cases respond to steroids, alkylating agents and IFNα, all with varying success rates [29]. The role of radiotherapy is still poorly understood, with some reports describing full resolution while others showed no response [18,22].

Although RDD in extremities had been described in a limited number of cases, this case highlighted the importance of better differentiation from more common malignancies. The painful symptoms experienced both before and after resection in the current case were uncharacteristic, as most patients with extranodal RDD had experienced pain-free results following resection. Furthermore, the development of bilateral disease in a different site had been only rarely reported [1,5,14,16].

## Conclusions

In summary, a defined treatment strategy for RDD has not been well described, given the rarity of the lesions and the difficulty in diagnosing them preoperatively. To date, surgical resection has proven most successful in preventing recurrences. Only one case of local recurrence for extranodal soft tissue RDD following surgical resection has been reported. However, as in the current case, bilateral disease presentation is also possible and requires close clinical follow-up. Given the rarity and indolence of RDD, surveillance cannot be endorsed; however, it is important to consider extranodal soft tissue RDD amongst the differential diagnosis of patients presenting with suspicious soft tissue masses. Future studies may help to elucidate the natural history of this disease process, as well as the possibility for malignant potential, thus permitting the development of more evidence-based treatment strategies. Until then, known RDD lesions should be excised using established surgical principles.

## Consent

Written informed consent was obtained from the patient for publication of this case report and any accompanying images. A copy of the written consent is available for review by the Editor-in-Chief of this journal.

## Abbreviations

H & E: Hematoxylin and eosin; IFN: Interferon; LCH: Langerhans cell histiocytosis; RDD: Rosai–Dorfman disease; SHML: Sinus histiocytosis with massive lymphadenopathy.

## Competing interests

The authors declare that they have no competing interests.

## Authors’ contributions

MK and LSS reviewed the literature and drafted the manuscript. RSC was clinically responsible for the patient’s care and revision of the manuscript. MD and MLS were responsible for the pathology. All authors read and approved the final manuscript.

## References

[B1] Kong Y Kong J Shi D Lu H Zhu X Wang J Chen Z Cutaneous Rosai–Dorfman Disease: a clinical and histopathologic study of 25 cases in China Am J Surg Pathol 2007 21 341 350 17325475 10.1097/01.pas.0000213387.70783.b6

[B2] Potts C Bozeman A Walker A Floyd W Cutaneous Rosai–Dorfman disease of the forearm: case report J Hand Surg Am 2008 33A 1409 1413 18929211 10.1016/j.jhsa.2008.04.003

[B3] Penna Costa AL Oliveira e Silva N Motta MP Athanazio RA Athanazio DA Athanazio PRF Soft tissue Rosai–Dorfman disease of the posterior mediastinum J Bras Pneumol 2009 35 717 720 19669012 10.1590/s1806-37132009000700015

[B4] Tan HY Kao LY Rosai–Dorfman disease manifesting as relapsing uveitis and subconjunctival masses Chang Gung Med J 2002 25 621 625 12479625

[B5] Yoon A Parisien M Feldman F Young-In Lee F Extranodal Rosai–Dorfman disease of bone, subcutaneous tissue and paranasal sinus mucosa with a review of its pathogenesis Skeletal Radiol 2005 34 653 657 10.1007/s00256-005-0953-4 16096753

[B6] Montgomery EA Meis JM Rosai–Dorfman disease of soft tissue Am J Surg Pathol 1992 16 122 129 10.1097/00000478-199202000-00004 1733347

[B7] Suster S Cartagena N Cabello-Inchausti B Robinson MJ Histiocytic lymphphagocytic panniculitis: an unusual extranodal presentation of sinus histiocytosis with massive lymphadenopathy (Rosai–Dorfman disease) Arch Dermatol 1988 124 1246 1249 10.1001/archderm.1988.01670080058019 3401030

[B8] Rasool MN Ramdial PK Osseous localization of Rosai–Dorfman disease J Hand Surg Br 1996 21B 349 350 8771474 10.1016/s0266-7681(05)80200-1

[B9] Govender D Chetty R Inflammatory pseudotumour and Rosai–Dorfman disease of soft tissue: a histological continuum? J Clin Pathol 1997 50 79 81 10.1136/jcp.50.1.79 9059366 PMC499722

[B10] Child FJ Fuller LC Salisbury J Higgins EM Cutaneous Rosai–Dorfman disease Clin Exp Dermatol 1998 23 40 42 10.1046/j.1365-2230.1998.00312.x 9667110

[B11] Quaglino P Tomasini C Novelli M Colonna S Bernengo MG Immunohistologic findings and adhesion molecule pattern in primary pure cutaneous Rosai–Dorfman disease with xanthomatous features Am J Dermatopathol 1998 20 393 398 10.1097/00000372-199808000-00013 9700380

[B12] Huang H Liang C Yang B Sung M Lin J Chen W Isolated Rosai–Dorfman disease presenting as peripheral mononeuropathy and clinically mimicking a neurogenic tumor: case report Surg Neurol 2001 56 344 347 10.1016/S0090-3019(01)00577-8 11750016

[B13] Stefanato CM Ellerin PS Bhawan J Cutaneous sinus histiocytosis (Rosai–Dorfman disease) presenting clinically as vasculitis J Am Acad Dermatol 2002 46 775 778 10.1067/mjd.2002.119565 12004323

[B14] Molina-Garrido MJ Guillen-Ponce C Extranodal Rosai–Dorfman disease with cutaneous periodontal involvement: a rare presentation Case Rep Oncol 2011 4 96 100 10.1159/000324760 21475597 PMC3072186

[B15] Shi X Ma D Fang K Cutaneous Rosai–Dorfman disease presenting as a granulomatous rosacea-like rash Chin Med J (Engl) 2011 124 793 794 21518580

[B16] Sodhi KS Suri S Nijhawan R Kang M Gautam V Rosai–Dorfman disease: unusual cause of diffuse and massive retroperitoneal lymphadenopathy Br J Radiol 2005 25 845 847 16110109 10.1259/bjr/23127241

[B17] Ensari S Selcuk A Dere H Perez N Dizbay Sak S Rosai–Dorfman disease presenting as laryngeal masses Kulak Burun Bogaz Ihtis Derg 2008 18 110 114 18628647

[B18] Pinto DCG Vidigal TA Castro B Santos BH DeSousa NJA Rosai–Dorfman disease in the differential diagnosis of cervical lymphadenopathy Bras J Otorrinolaringol 2008 74 632 635 10.1590/S0034-72992008000400025 PMC9442606 18852995

[B19] Levine PH Jahan N Murari P Manak M Jaffe ES Detection of human herpesvirus 6 in tissues involved by sinus histiocytosis with massive lymphadenopathy (Rosai–Dorfman disease) J Infect Dis 1992 166 291 295 10.1093/infdis/166.2.291 1321861

[B20] Luppi M Barozzi P Garber R Maiorana A Bonacorsi G Artusi T Trovato R Marasca R Torelli G Expression of human herpesvirus-6 antigens in benign and malignant lymphoproliferative diseases Am J Pathol 1998 163 815 823 9736030 10.1016/S0002-9440(10)65623-4 PMC1853007

[B21] Stebbing C van der Walt J Ramadan G Inusa B Rosai–Dorfman disease: a previously unreported association with Sickle cell disease BMC Clin Path 2007 7 3 10.1186/1472-6890-7-3 17407562 PMC1855345

[B22] Moore J Zhao X Nelson E Concomitant sinus histiocytosis with massive lymphadenopathy (Rosai–Dorfman disease) and diffuse large B-cell lymphoma: a case report J Med Case Reports 2008 2 70 10.1186/1752-1947-2-70 PMC2270859 18321383

[B23] Woodcock R Madell J Lipper M Sinus histiocytosis (Rosai–Dorfman disease) of the suprasellar region: MR imaging findings – a case report Radiology 1999 213 808 810 10580957 10.1148/radiology.213.3.r99dc30808

[B24] Lu D Estalilla OC Manning JT Medeiros J Sinus histiocytosis with massive lymphadenopathy and malignant lymphoma involving the same lymph node: a report of four cases and review of the literature Mod Pathol 2000 13 414 419 10.1038/modpathol.3880071 10786808

[B25] Shea CR Elston DM Langerhans Cell Histiocytosis http://emedicine.medscape.com/article/1100579-overview

[B26] Kemson R Rouse R *Inflammatory Myofibroblastic Tumor* Surgical pathology criteria 2008 Stanford, CA: Stanford School of Medicine http://surgpathcriteria.stanford.edu/softfib/inflammatory_myofibroblastic_tumor/

[B27] Sabel MS Greenfield LJ, Mulholland MW From sarcomas of bone and soft tissue Greenfield’s Surgery: Scientific Principles and Practice 2011 5 Philadelphia, PA: Wolters Kluwer Health/Lippincott William and Wilkins 2151 2176

[B28] Konishi E Ibayashi N Yamamoto S Scheithauer BW Isolated intracranial Rosai–Dorfman disease (sinus histiocytosis with massive lymadenopathy) Am J Neuroradiol 2003 24 515 518 12637307 PMC7973600

[B29] Podberezin M Angeles R Guzman G Peace D Gaitonde S Primary pancreatic sinus histiocytosis with massive lymphadenopathy (Rosai–Dorfman disease): an unusual extranodal manifestation clinically simulating malignancy Arch Pathol Lab Med 2010 134 276 278 20121618 10.5858/134.2.276

